# Effects of respiratory muscle training on parasympathetic activity in diabetes mellitus

**DOI:** 10.1590/1414-431X2020e10865

**Published:** 2021-05-17

**Authors:** C.S.C. Trevisan, A.S. Garcia-Araújo, A.C.G.O. Duarte, V.O. Furino, T.L. Russo, A. Fujimoto, H.C.D. Souza, R.B. Jaenisch, R. Arena, A. Borghi-Silva

**Affiliations:** 1 Departamento de Fisioterapia, Universidade Federal de São Carlos, São Carlos, SP, Brasil; 2 Departamento de Educação Física e Motricidade Humana, Universidade Federal de São Carlos, São Carlos, SP, Brasil; 3 Departamento de Ciências da Saúde, Curso de Fisioterapia, Faculdade de Medicina de Ribeirão Preto, Universidade de São Paulo, Ribeirão Preto, SP, Brasil; 4 Departamento de Fisioterapia e Reabilitação, Universidade Federal de Santa Maria, Santa Maria, RS, Brasil; 5 Department of Physical Therapy, College of Applied Health Sciences, University of Illinois at Chicago, Chicago, IL, USA

**Keywords:** Diabetes mellitus, Exercise, Respiratory muscle training, Autonomic control

## Abstract

This study verified the effects of respiratory muscle training (RMT) on hemodynamics, heart rate (HR) variability, and muscle morphology in rats with streptozotocin-induced diabetes mellitus (DM). Thirty-six male Wistar rats were randomized into 4 groups and 34 completed the study: i) sham-sedentary (Sham-ST; n=9); ii) sham-RMT (Sham-RMT; n=9); iii) DM-sedentary (DM-ST; n=8); and iv) DM-RMT (DM-RMT; n=8). Hemodynamics were assessed by central cannulation, and R-R intervals were measured by electrocardiogram. In addition, the effects of RMT on the cross-sectional area of the diaphragm, anterior tibial, and soleus muscles were analyzed. The induction of DM by streptozotocin resulted in weight loss, hyperglycemia, reduced blood pressure, and attenuated left ventricular contraction and relaxation (P<0.05). We also observed a decrease in root mean square of successive differences between adjacent RR intervals (RMSSD) index and in the cross-sectional area of the muscles assessed, specifically the diaphragm, soleus, and anterior tibial muscles in diabetic rats (P<0.05). Interestingly, RMT led to an increase in RMSSD in rats with DM (P<0.05). The induction of DM produced profound deleterious changes in the diaphragmatic and peripheral muscles, as well as impairments in cardiovascular hemodynamics and autonomic control. Nevertheless, RMT may beneficially attenuate autonomic changes and improve parasympathetic modulation.

## Introduction

Diabetes mellitus (DM) is a risk factor for cardiovascular diseases, and impairments in the peripheral nervous system are frequently observed in this condition (1). Autonomic neuropathy is a complication that affects autonomic modulation and reduces heart rate variability (HRV) (2). Sympathetic dysfunction in patients with DM involves denervation (distal) and hyperinervation (proximal) of the left ventricle, causing arrhythmias (3). For this reason, autonomic neuropathy is associated with high morbidity and mortality in individuals with DM (4).

Additionally, deleterious muscular changes are present due to diabetic myopathy. It includes sarcomere rupture and disturbances in calcium metabolism. It decreases muscle oxidative capacity and protein synthesis by insulinopenia. On the other hand, it increases intramuscular lipids, proteolysis, and loss of muscle tissue regeneration capacity (5–7). This process results in reduced muscular strength and functional capacity (8).

Respiratory muscle training (RMT) in patients with DM has been shown to improve hemodynamics, functional capacity, inspiratory muscle strength, as well as to increase diaphragm muscle thickness (9,10). In a study by Silva et al. (11), the authors found that RMT in elderly people with insulin resistance resulted in an increase in GLUT4 protein, an important cellular transporter of glucose. Moreover, RMT in rats with heart failure improved hemodynamic function and sympathovagal activity (12).

Although some studies have shown RMT positively impacts hemodynamic function and HRV in an animal heart failure model (12), the potential effects of RMT in the presence of DM are still poorly understood. Therefore, the present study tested the hypothesis that a DM model in rats induces both deleterious central and peripheral changes. It aimed to analyze if RMT improves hemodynamic function, HRV, and muscle characteristics.

## Material and Methods

The experimental protocol of this study was approved by the Ethics Committee on Animal Use of the Federal University of São Carlos (CEUA/UFSCAR-1778140618), and was conducted in accordance with accepted standards of animal care as outlined in the Ethical Guidelines. Thirty-six male Wistar rats with approximately 90 days of age (170-270 g), from the UFSCar vivarium, were housed in a grouped cage (n=3/cage), temperature-controlled environment (21°C), with water and food *ad libitum* and a 12-h light/dark cycle.

### Experimental groups

Before the allocation of animals in groups, animals were acclimatized to the environment. Upon arriving from the vivarium, before any procedure, the animals were randomly assigned a number (1 to 36) on the tail with a pen. Then, researchers randomly allocated nine animals to one of four groups: Sham with RMT (Sham RMT), Sham without RMT (Sham ST (sedentary)), DM with RMT (DM RMT), and DM without RMT (DM ST).

### Diabetes mellitus induction

Glycemia and weight of animals were measured once a week for two weeks before diabetes induction. With a 12-h fast, the rats were induced to DM by receiving a single intraperitoneal injection of 50 mg/kg of streptozotocin (Sigma-Aldrich, USA) diluted in 1 mL of sodium citrate buffer (pH 4.5). The Sham groups received the same volume of sodium citrate (pH 4.5) by intraperitoneal injection. On the fifth day after induction, DM was confirmed by hyperglycemia >250 mg/dL (13). Glycemia was determined after analyzing the blood from the tail vein, using a portable glucometer (Accu-Check; Roche Diagnostic, USA).

### Respiratory muscle training protocol

**Figure 1:**
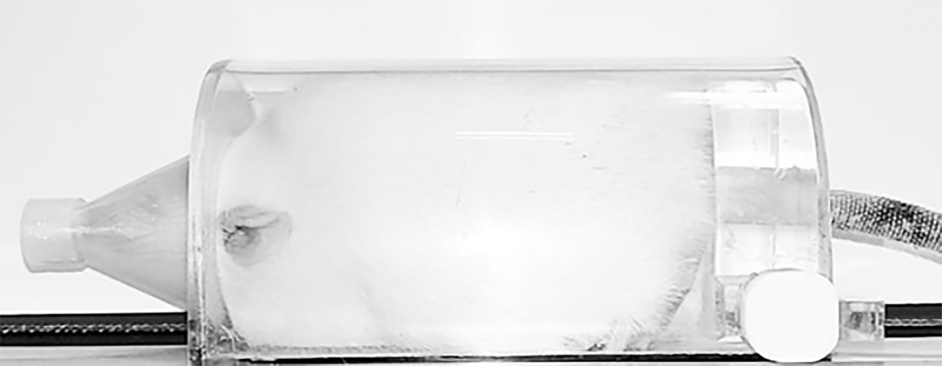
Acrylic cylinder for the ventilatory muscle training of rats.

The RMT protocol was performed during the night, 30 min/day, 5 days/week, for 6 weeks. The load was generated using a breathing tube (acrylic cylinder) with an orifice (Figure 1). The head of the animal was placed on the end of the equipment (cone) providing breathing resistance training as reported by Jaenisch et al. (12). The initial diameter of the hole was 0.8 mm and it was reduced to 0.7, 0.6, 0.55, 0.45 mm in the following weeks. During the final week, it was 0.3 mm, which corresponded to the maximum resistance for this training (12,14). The group without RMT was submitted to the same conditioning, but without resistance.

### Cannulation of animals and placement of electrocardiogram electrodes

After the training protocol, the rats were anesthetized intraperitoneally with ketamine (80 mg/kg) and xylazine (12 mg/kg) and were then placed on a heated platform to record the electrocardiogram (ECG). Three electrodes were attached in the subcutaneous region, the positive and negative (proximal) in the pectoral region and the neutral (distal) in the anterior region of the right paw. The ECG was recorded for 5 minutes, followed by cannulation of the right carotid artery, using a polyethylene catheter (PE50) to record arterial and ventricular pressures (15).

### Blood pressure and left ventricular pressure recording

A signal acquisition system (PowerLab 8/35 Pro, AD Instruments, Australia) was connected to a signal amplifier (Bridge Amp FE221, AD Instruments) to record arterial and ventricular pressures. Data were recorded at 1000 points per second. The setting parameters were as follows: 1) Channel 1 for ECG: with sample rate at 1 k/s, range at 5 mV, connected to Bio Amplifier, and units in mV; 2) Channel 2 for pressure measurements: with sample rate at 1 k/s, range at 50 mV, connected to Bridge Amplifier, and units in mmHg; and 3) Channel 3 with a derivation of Channel 2 for HR: with sample rate at 1 k/s, range at 2 mV. A pressure transducer (MLT844; AD Instruments) was connected with one of its ends to the signal amplifier and the other end was coupled to an intermediate pressurized water seal system. The latter was necessary for system calibration and adequate pressure recording. The pressure calibration for the transducer was performed using a pressure gauge, where the pressures were recorded, from 0 to 250 mmHg.

Central arterial pressure was recorded for 5 min through the cannula in the right carotid. Then, the cannula was introduced and positioned in the left ventricle (LV), recording for 5 min more. The position of the cannula was determined by observing the ventricular pressure wave characteristic (15).

### Muscle histology

The animals were euthanized by means of an intravenous overdose infusion of pentobarbital anesthetic (180 mg/kg). Diaphragm, soleus, and anterior tibial muscles were dissected, weighed, frozen in liquid nitrogen, and stored at -80°C (16) for further analysis. Transverse sections of the muscles (10 μm thick) were performed in the cryostat (CM 1850 UV; LEICA^®^, Germany) at a temperature of -30°C. The sections were stained with toluidine blue and analyzed under light microscopy (Axiovision 3.0.6 SP4, Carl Zeiss, Germany). Four microphotographs of the central portion of the sections at 20× magnification were taken. A blind evaluator from the research group measured the cross-sectional areas of 100 fibers manually, at random, using ImageJ software, version 1.43u, (National Institutes of Health, USA) (17).

### Sample size calculation, data extraction, and statistical analysis

A minimum of 8 rats per group was determined by Gpower software (Germany), using the HF variable as in the study by Jaenisch (18), to achieve a statistical power of 80% (β=0.20), with α=0.05. However, we used a sample N of 9 to account for a loss of 10% for each group.

Analysis also used the Labchart 8 program (AD Instruments) associated with the data acquisition system Bridge Amp FE221 (AD Instruments) (16). The hemodynamic variables were determined using the average of the most stable 5-min time series of the signal. HRV was calculated based on the R-R intervals (R-Ri) of ECG. Hemodynamic measurements were: 1) systolic pressure; 2) diastolic pressure; 3) dicrotic pressure; 4) average pressure; 5) pulse pressure; 6) ejection duration; 7) non-ejection duration; 8) cycle duration; 9) heart rate; 10) peak hour; 11) average diastolic pressure; 12) maximum pressure; 13) minimum pressure; 14) final diastolic pressure; 15) mean pressure; 16) maximum-minimum pressure; 17) systolic duration; 18) diastolic duration; 19) cycle duration; 20) heart rate; 21) ventricular contraction; 22) isovolumetric relaxation period; and 23) pressure time index (15). The following R-Ri were analyzed using 5-min samples, considering the most stable acquisition stretches, by graphical visual analysis: 1) linear methods in the time domain: heart rate (HR); 2) average of RR intervals (average RR); 3) median of RR intervals (median RR); 4) square root of the variance of the entire time series (SDRR); and 5) root mean square of successive differences between adjacent RR intervals (RMSSD). The following calculations were made for the frequency domain: 1) low frequency (LF (nu)); 2) high frequency (HF (nu)); and 3) low frequency/high frequency (LF/HF) ratio. Nonlinear calculations were made, as follows: 1) the standard deviation of the instant variability of the beat-to-beat interval (SD1) and 2) the long-term variability of continuous RRi (SD2) (19).

The data were analyzed using the SigmaPlot software (Systat, USA). Normality tests were performed by Shapiro-Wilk and data are reported as means±SD (parametric) or as median, as well as the minimum and maximum values (non-parametric). To compare the effects between the groups and the intervention, one-way ANOVA test was applied, followed by the Tukey's *post hoc* test. Statistical significance was set at P<0.05 for all analyses.

## Results

RMT was started with 36 animals. During 6 weeks of training, 2 animals died - 1 animal in the DM RMT group and 1 in the DM ST group, and 34 animals completed the experiment. Body mass, muscle and heart weights, and baseline glycemia data are reported in Table 1. We observed that DM significantly reduced the heart weight compared to all other groups (P<0.05). It is noteworthy that in the DM RMT group, the heart weight was higher than in the DM ST group (P<0.05). In addition, DM groups showed reduced diaphragm, soleus, and anterior tibial muscle weights compared to the other groups (P<0.05). Notwithstanding, RMT did not attenuate the reduction in muscle weight of these muscle groups compared to DM ST (P>0.05) (Table 1).

**Table 1:** Measurement of weight, blood glucose, and muscle fibers between groups of rats with streptozotocin-induced diabetes mellitus (DM), sedentary (ST) or treated with respiratory muscle training (RMT), and controls (Sham).

Group	Sham RMT (n=9)	Sham ST (n=9)	DM RMT (n=8)	DM ST (n=8)	F	P
Basal weight (g)	181.4±9.0	186.4±8.3	182.8±17.1	175.8±22.5	0.71	0.55
Post-induction weight (g)	244.7±15.6	249.2±9.7	217.3±30.6	222.8±26.2	4.48	0.01
Post-RMT weight (g)	418.0±32.4	410.5±27.0	324.2±62.7€α	319.1±69.0#*	9.70	<0.001
Basal glucose (mg/dl)	130.4±7.1	131.5±20.7	127.7±11.5	127.6±15.9	0.15	0.93
Post-induction glucose (g)	119.4±36.3	136.7±21.4	488.7±132.1€α	460.0±73.9#*	58.41	<0.001
Glucose post-RMT (mg/dL)	109.7±9.3	111.6±13.9	477.0±149.6€α	451.3±100.4#*	46.23	<0.001
Heart weight (g)	1.5±0.1	1.4±0.1	1.3±0.1	1.2±0.2#*†	5.73	0.003
Diaphragm weight (g)	0.8±0.1	0.8±0.1	0.6±0.1€α	0.5±0.1#*	12.11	<0.001
Tibialis anterior weight (g)	0.7±0.0	0.8±0.1	0.5±0.1€α	0.5±0.1#*	10.69	<0.001
Soleus weight (g)	0.2±0.0	0.2±0.0	0.1±0.0α	0.1±0.0#	3.23	0.03
Diaphragm (µm)	284.2±170.0	304.1±88.7	196.1±30.1	163.5±53.4*	3.80	0.02
Tibialis anterior (µm)	330.3±94.9	378.5±39.6	228.4±55.5€α	257.3±49.6*	8.96	<0.001
Soleus (µm)	357.5±62.7	424.9±87.02	285.7±53.2€	290.4±46.6*	8.00	<0.001

Data are reported as means±SD. ^#^P<0.05 compared with Sham RMT; *P<0.05 compared with Sham ST; ^†^P<0.05 compared with DM RMT; ^€^P<0.05 compared with Sham ST; ^α^P<0.05 compared with Sham RMT (one-way ANOVA with Tukey's multiple comparisons *post hoc* test).

### Histological changes in muscle fibers

The diaphragm, tibialis anterior, and soleus muscles of DM ST rats had a smaller cross-sectional area compared to Sham ST rats (P<0.05). The tibialis anterior and the soleus muscles of DM RMT rats also showed a smaller cross-sectional area compared to Sham ST (P<0.05). Moreover, the tibialis anterior muscle of DM RMT rats presented a smaller cross-sectional area compared to Sham RMT rats (P<0.05) (Table 1).

### Hemodynamic variables

The derivatives (channel 3) of the blood pressure record of the rats showed a lower systolic pressure in DM ST rats, compared to the Sham ST and Sham RMT groups (P<0.05). Diastolic and mean pressure also showed significantly lower values in the DM ST group, compared to Sham RMT rats (P<0.05). No difference in relation to the other groups was observed in the DM RMT group. Heart rate was lower in rats with DM and Sham ST rats, compared to Sham RMT (P<0.05) (Table 2).

**Table 2:** Hemodynamic variables derived from the blood pressure records of rats with streptozotocin-induced diabetes mellitus (DM), sedentary (ST) or treated with respiratory muscle training (RMT), and controls (Sham).

Group	Sham RMT (n=9)	Sham ST (n=9)	DM RMT (n=8)	DM ST (n=8)	F	P
HR (bpm)	219.5±46.4	212.1±29.7	196.7±35.8	210.5±33.5	0.55	0.65
Average RR (ms)	299.8±54.1	291.4±45.8	317.2±42.6	295.1±40.4	0.51	0.68
Median RR (ms)	311.7±50.1	287.6±47.5	316.5±40.5	295.1±35.0	0.81	0.49
SDRR (ms)	44.7±30.1	25.2±15.5	26.5±21.7	22.7±16.6	1.8	0.15
RMSSD (ms)	24.01±12.6	16.7±10.9	20.6±14.1	7.9±5.8^#^†	3.0	0.04
LF (nu)	19.9±10.7	20.8±14.3	16.6±6.6	16.1±8.2	0.42	0.74
HF (nu)	62.2±9.9	57.3±14.4	61.3±11.1	58.0±15.7	0.30	0.82
LF/HF	0.3±0.2	0.4±0.3	0.3±0.1	0.2±0.1	0.56	0.65
SD1 (ms)	15.9±8.9	13.02±7.7	12.1±10.8	9.9±5.9	0.86	0.47
SD2 (ms)	60.8±4	33.1±21.4	34.5±29.3	29.5±22.8	1.98	0.14
Systolic pressure (mmHg)	133.06±22.4	129.8±12.8	114.7±23.2	109.5±13.1^#^*	3.20	0.03
Diastolic pressure (mmHg)	106.7±18.6	103.9±14.1	85.2±17.8	79.3±17.2^#^	5.39	0.004
Dicrotic notch pressure (mmHg)	114.7±21.0	103.1±22.7	93.4±23.07	86.2±18.9	2.29	0.10
Mean pressure (mmHg)	120.9±19.9	117.4±13.5	99.9±20.01	94.9±14.8^#^	4.63	0.009
Pulse pressure (mmHg)	26.2±5.7	25.8±5.5	29.4±7.8	30.2±11.3	0.67	0.58
Ejection duration (s)	1.0±0.8	0.6±0.5	0.5±0.3	0.4±0.2	1.38	0.27
Non-ejection duration (s)	0.6±0.5	0.6±0.5	0.3±0.2	0.3±0.1	0.89	0.46
Cycle duration (s)	0.9±1.1	0.4±0.3	0.3±0.06	0.3±0.1	1.91	1.15
Time to peak (s)	0.3±0.4	0.1±0.1	0.1±0.03	0.1±0.03	2.28	0.10
Mean diastolic pressure (mmHg)	118.5±17.3	105.9±26.2	95.6±18.09	93.3±15.2	2.29	0.10

Data are reported as means±SD. HR: heart rate; RR: RR intervals; SDRR: square root of the variance of the entire time series; RMSSD: root mean square of successive squared differences between adjacent RR intervals; LF: low frequency; HF: high frequency; LF/HF: low frequency/high frequency ratio; SD1: standard deviation of the instant variability of the beat-to-beat interval; SD2: long-term variability of continuous RRi. ^#^P<0.05 compared with Sham RMT; ^†^compared with DM RMT; *P<0.05 compared with Sham ST (one-way ANOVA with Tukey's multiple comparisons *post hoc* test).

As for ventricular function, a longer period of left ventricular contraction was observed in DM ST rats compared to Sham RMT (P<0.05). Additionally, a longer period of relaxation occurred in DM ST rats compared to Sham ST or Sham RMT rats (P<0.05) (Table 3).

**Table 3:** Variables of ventricular function and cardiac chamber pressures.

Group (n)	Sham RMT (n=9)	Sham ST (n=9)	DM RMT (n=8)	DM ST (n=8)	F	P
Max pressure (mmHg)	114.3±28.4	106.0±30.9	101.9±20.6	116.5±22.1	0.46	0.71
Min pressure (mmHg)	13.2±8.7	10.3±6.5	10.001±7.1	7.8±6	0.77	0.52
EDP (mmHg)	15.6±8.6	13.08±5.2	16.1±8.6	14.2±4.7	0.23	0.88
Mean pressure (mmHg)	51.9±13.3	48.1±12.7	47.9±7.1	54.8±7.4	0.61	0.62
Max-Min pressure (mmHg)	101.1±27.4	95.7±31.9	91.9±27.7	108.6±26.2	0.43	0.73
Systolic duration (s)	0.1±0.0	0.1±0.0	0.1±0.0	0.1±0.0	1.89	0.16
Diastolic duration (s)	0.1±0.0	0.1±0.0	0.1±0.0	0.2±0.0	11.59	<0.001
Cycle duration (s)	0.2±0.0	0.2±0.0	0.3±0.1	0.2±0.0	0.43	0.73
Heart rate (bpm)	216.7±40.9	226.3±37.9	207.8±41.1	211.4±26.9	0.28	0.84
Max dP/dt (mmHg/s)	2233.0±929.5	2760.4±1261.4	2771.9±1176.9	3989.1±769.6^#^	3.95	0.02
Average dP/dt (mmHg/s)	-1105.3±540.4	-1167.6±651.6	-1370.6±463.1	-1982.9±547.3^#^*	3.80	0.02
Tau (s)	0.04±0.0	0.05±0.02	0.2±0.3	0.06±0.1	2.07	0.13
Pressure Time Index (mmHg)	10.5±3.3	9.7±3.1	10.9±2.1	13.2±3.2	1.65	0.20

Data are reported as means±SD. RMT: respiratory muscle training; ST: sedentary; DM: diabetes mellitus; EDP: end diastolic pressure; Max dP/dt: ventricular contraction. ^#^P<0.05 compared with Sham RMT; *P<0.05 compared with Sham ST (one-way ANOVA with Tukey's multiple comparisons *post hoc* test).

### Changes in HRV

**Figure 2:**
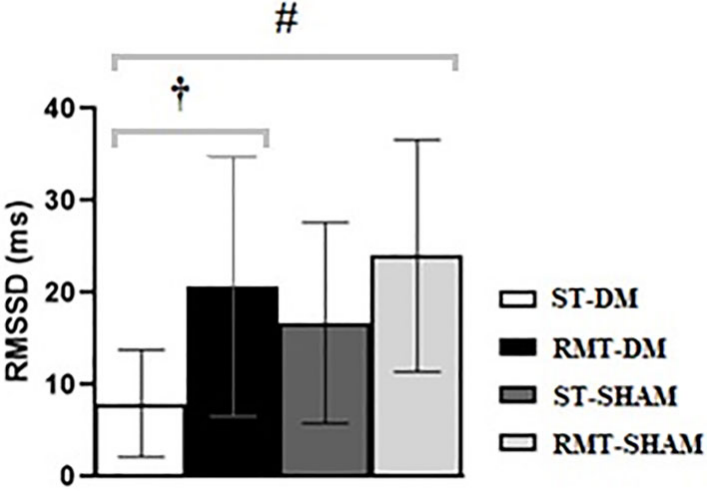
Comparison of root mean square of successive differences between adjacent RR intervals (RMSSD) between groups of rats with streptozotocin-induced diabetes mellitus (DM) or controls (Sham). ^#†^P<0.05 (ANOVA test followed by Tukey's *post hoc* test). RMT: respiratory muscle training; ST: without training.

Diabetes mellitus produced a significant reduction in the RMSSD index. Interestingly, in both RMT trained groups (Sham RMT and DM RMT groups), the RMSSD index was higher, showing an improvement of parasympathetic modulation compared to DM ST animals (P<0.05) (Figure 2).

## Discussion

The results of this study demonstrated that streptozotocin diabetes induced metabolic, hemodynamic (i.e., arterial and ventricular), autonomic, and muscular disorders. We also found that RMT may beneficially attenuate HR and autonomic changes and improve parasympathetic modulation.

In relation to hemodynamic measurements, DM produced significant impairments by decreasing systolic, diastolic, and mean blood pressure. Conversely, HR increased and poor ventricular performance was observed. This decrease in the baseline blood pressure in DM rats has been studied previously (20,21), and is associated with a reduction in ventricular performance (20). In our investigation, the DM ST group demonstrated less LV contractility compared to RMT sham. We also observed a decrease in LV compliance both in Sham RMT and Sham ST, which also corroborates previous studies (22,23).

The sympathetic dysfunction produced by DM is explained by the distal denervation and proximal hyperinnervation of the left ventricle. The electrical stability and myocardial blood flow change, leading to increased arrhythmia risk (3). In this context, Otake et al. (24) reported that impaired neural remodeling may play a crucial role in increasing vulnerability to atrial fibrillation as a consequence of DM.

In the study by Li et al. (25) that evaluated HRV in DM rats induced by streptozotocin, it was shown that sympathetic modulation increased while parasympathetic modulation decreased. These findings were explained by the intense remodeling that occurs with the progression of the disease in the autonomic nerves produced by the model. Other studies have also demonstrated this decrease in parasympathetic tone and a predominance of sympathetic changes in rats with DM by streptozotocin (20,26).

Our findings revealed that RMT lowered HR in the DM RMT group compared to Sham ST and potentiated parasympathetic modulation. This was demonstrated by an increased RMSSD index, reversing DM-induced pathophysiology in DM rats. In a heart failure induction model, Jaenisch et al. (18) used the same RMT protocol and demonstrated similar results - a favorable modulation in autonomic tone quantified by the parasympathetic HRV index. Moreover, in individuals with DM, RMT was also effective in improving respiratory strength and autonomic tone using an 8-week protocol (9).

These findings may be explained by the potential effects of RMT observed through changes in the breathing pattern and activation of the interaction of physiologic reflexes: 1) central and peripheral chemoreflex (stimulated by changes in CO_2_ and O_2_); 2) metabolic reflex (activated by the accumulation of metabolites); and 3) baroreflex (pressure sensitive). Increased ventilation stimulates pulmonary stretch receptors and prevents an increase in inspiratory volume. Furthermore, increased blood pressure stimulates baroreceptors that reduce sympathetic nerve activity and increase vagal activity, which leads to a reduction in blood pressure and HR (10,20,27,28).

The RMT DM group presented a greater cardiac weight compared to the DM ST group. Nevertheless, RMT did not prevent a decrease in muscle mass in DM rats compared to the Sham ST group, as demonstrated by the smaller muscle fiber cross-sectional area in the diaphragm, soleus, and tibialis anterior muscles.

A consensus on an optimal approach to RMT has not been reached at this time. Further research could address this issue using different protocols. With respect to patients with DM, future trials on RMT should analyze the benefit of metabolic outcomes such as glycemic control. In previous research, subjects with insulin resistance showed reduced glucose and insulinemia following a 12-week RMT protocol using a 40% maximum inspiratory pressure (PImax) load (11). In elderly people with hyperglycemia, an 8-week RMT protocol ameliorated glucose metabolism and delayed a possible evolution to diabetes (29). Individuals with metabolic syndrome showed no decrease in glucose after a 7-day RMT protocol (15 min, 3 times a day) at a 30% load of PImax (30). Lastly, a previous study observed that individuals with type 2 DM demonstrated a 40% reduction in glucose levels with a single high-strength RMT load (60% PImax) (31).

Bisschop et al. (14), using an 8-week low to moderate intensity inspiratory load protocol, found an increase in the dimensions of diaphragmatic fibers in rats. Conversely, we did not observe this in our 6-week protocol. Perhaps, due to the fact that we had untreated diabetic animals, the animals had a marked loss of muscle mass and the 6-week protocol was not able to promote spherical adaptations in these animals. Notably, the present study did not use drug treatment in animals for hyperglycemic control. Li et al. (25) demonstrated that the HRV index can be normalized to different degrees with the use of insulin and meticobal. Nonetheless, these indexes did not return to normal after treatment. In the present study, we observed that RMT alone was an important strategy for parasympathetic modulation, an important marker of cardiovascular risk and death in patients with DM (32).

The findings of this study are subject to certain limitations. Firstly, the use of drug induction is not the most appropriate method to represent DM in humans. However, the streptozotocin model is widely used despite the sample loss, due to the inability of some animals to survive the approach. Another widely discussed point is the toxicity of streptozotocin to organs, including muscle tissue. Secondly, the RMT training protocol used in the current study differs from RMT in humans. In addition, despite the standardization of the orifices, it was not possible to monitor the load imposed during the animals' breathing. This pressure control would allow the standardization of the load imposed on training. Finally, although HRV is commonly measured by ECG using sedation of animals with ketamine and xylazine, it is important to note that the sedation may attenuate cardiac autonomic indices (33). 

In conclusion, DM induced by streptozotocin resulted in loss of cardiac mass, altered hemodynamics, impaired autonomic tone, and caused a longer period of contraction and relaxation of the LV. Yet, interestingly, RMT resulted in improvement in parasympathetic modulation in rats with streptozotocin-induced DM, supporting the potential applications of RMT in the DM population.
